# The cardiac toxicity of PAMAM dendrimer drug delivery systems can be attenuated with the adjunct use of cardioprotective agents

**DOI:** 10.17305/bb.2024.10735

**Published:** 2024-09-12

**Authors:** Saghir Akhtar, Fawzi Babiker, Aisha Al-Kouh, Ibrahim F Benter

**Affiliations:** 1 College of Medicine, QU Health, Qatar University, Doha, Qatar; 2 Faculty of Medicine, Kuwait University, Kuwait City, Kuwait; 3 Faculty of Pharmacy, Final International University, Kyrenia, North Cyprus

**Keywords:** Ischemia/reperfusion, cardiac injury, cardiotoxicity, PAMAM dendrimer, nanoparticle, Losartan, epidermal growth factor, nitric oxide, S-nitroso-N-acetylpenicillamine

## Abstract

Polyamidoamine (PAMAM) dendrimer nanoparticles are efficient drug delivery vectors with potential clinical applications in nanomedicine. However, PAMAMs can compromise heart function, and strategies to mitigate cardiotoxicity would be beneficial. In this study, we investigated whether the adjunct use of three key cardioprotective agents could prevent cardiac injury induced by a seventh-generation cationic PAMAM dendrimer (G7). Isolated rat hearts were subjected to ischemia and reperfusion (I/R) injury in the presence or absence of G7 or the cardioprotective agents Losartan, epidermal growth factor (EGF), or S-nitroso-N-acetylpenicillamine (SNAP). I/R injury significantly compromised cardiac function, in terms of left ventricular (LV) hemodynamics, contractility, and vascular dynamics, which were markedly improved (*P* < 0.05) by the administration of Losartan, EGF, or SNAP alone, confirming their cardioprotective effects. The administration of G7 significantly worsened cardiac function recovery following I/R (*P* < 0.05). G7-induced impairments in cardiac and vascular dynamics were significantly improved by co-administration of Losartan, EGF, or SNAP. Treatment with G7 also significantly increased cardiac enzyme levels and infarct size, both of which were markedly reduced upon co-infusion of Losartan, EGF, or SNAP (*P* < 0.05). Thus, G7 deteriorates the recovery of cardiac function in isolated hearts subjected to I/R injury, which can be rescued by co-administration of Losartan, EGF, or SNAP. These findings enhance our understanding of PAMAM dendrimer nanotoxicology in the mammalian heart and suggest that the adjunct use of cardioprotective agents is an effective strategy for mitigating the cardiotoxicity of these dendrimers and potentially other drug delivery systems.

## Introduction

The hyperbranched, multivalent polyamidoamine (PAMAM) family of dendrimers are nano-sized nanoparticulate drug delivery systems with well-defined molecular architecture and tunable surface chemistry, offering potential applications in nanomedicine [1–3]. However, while PAMAM dendrimers improve cellular and tissue drug delivery, they can also exhibit intrinsic toxicological and biological actions that, in some cases, modulate key signaling pathways crucial for organ function [2, 4–13]. The net consequences of PAMAM-cell interactions *in vivo* can be either beneficial or detrimental, depending on the physicochemical properties of the dendrimers, such as surface charge, molecular weight, size, or generation, as well as the affected cell or tissue type [2, 4, 14]. For example, we previously reported on the beneficial vascular effects of PAMAM dendrimers in improving diabetes-induced vascular dysfunction [8]. However, the direct effects of PAMAM dendrimers on the heart and cardiac hemodynamics, as well as pharmacological strategies to counter any adverse effects, are not completely understood.

Higher generation PAMAMs are known to accumulate in the heart, particularly in ischemic regions of cardiac tissue [15]. Consistent with this finding, we showed that PAMAMs, especially higher generations like cationic amino-terminal group G6 and G7, could impair cardiac recovery from ischemia/reperfusion (I/R) injury, with overall cardiotoxicity being significantly dependent on dendrimer generation and surface charge [16, 17]. Cationic PAMAM dendrimers could also completely abrogate the beneficial/protective effects of postconditioning in rat hearts [17]. Therefore, strategies to mitigate the cardiotoxicity of these important drug delivery systems are highly desirable to ensure their potential safety in clinical settings. We recently reported that PAMAM-induced cardiotoxicity might be mitigated through co-administration of the heptapeptide angiotensin (1–7), termed Ang–(1–7), a member of the renin-angiotensin system (RAS) [16]. In the present study, we extend our previous findings to investigate whether other cardioprotective agents with differing mechanisms of action, such as Losartan (an angiotensin II type 1 receptor (AT1R) blocker), epidermal growth factor (EGF, a ligand for the EGF receptor tyrosine kinase), or S-nitroso-N-acetylpenicillamine (SNAP, a nitric oxide [NO] donor), could also prevent PAMAM-induced cardiac dysfunction following I/R injury.

Losartan is a well-established cardioprotective drug used clinically to treat various cardiovascular diseases [18]. It is a selective antagonist of type 1 angiotensin II receptors (AT1R), which are part of the detrimental arm of the RAS (e.g., [19]). Losartan is known to protect the heart in I/R injury [20, 21], particularly in the diabetic heart [22–24]. However, its role in protecting the heart from PAMAM dendrimer-induced injury is not known and is the subject of this study.

EGF is a ligand for receptors from the EGFR/ErbB family of receptor tyrosine kinases, which play important roles in the cardiovascular system, and it is a proven cardioprotective agent [22, 25]. We previously reported that EGF administration and subsequent activation of EGFR signaling protected the diabetic heart from I/R injury [22, 26]. Additionally, EGF was recently reported to reduce infarct size and myocardial apoptosis due to I/R injury [27]. With these cardioprotective characteristics, EGF appears to be a suitable candidate for the potential mitigation of PAMAM dendrimer-induced cardiac dysfunction.

Endothelial nitric oxide synthesis (eNOS) plays an essential role in protecting the heart from I/R injury [28–33]. Blocking nitric oxide (NO) production has been reported to result in the loss of cardioprotection from I/R injury [31], whereas NO induced during I/R protects the heart [32]. Indeed, NO protects the myocardium from I/R by improving vascular blood flow [33]. Additionally, NO is a potent scavenger of reactive oxygen species (ROS), which are known to harm the heart during I/R [34]. NO can be released *in vivo* using NO donors, such as organic nitrates, which are routinely used clinically to treat ischemic heart disease (e.g., angina pectoris) [35, 36]. In this study, we used the NO donor SNAP, which is known to be cardioprotective following I/R injury [37, 38], to investigate the role of NO in mitigating the cardiotoxicity of cationic G7 PAMAM dendrimers. To the best of our knowledge, this investigation represents the first time that these diverse cardioprotective agents have been shown to protect the heart from the cardiotoxic effects of PAMAM dendrimers.

## Materials and methods

Male Wistar rats weighing between 250 and 350 g were sourced from the Animal Resources Center at Kuwait University, Kuwait, with study approval obtained from the Health Science Center, Kuwait University Animal Ethics Committee, in accordance with the EU Directive 2010/63/EU for animal experiments. The rats were housed in plastic cages, two per cage, maintained under controlled conditions of temperature (21 ^∘^C–24 ^∘^C), a 12-h (7 a.m.–7 p.m.) light/dark cycle, and humidity (50%), with ad libitum access to food and water. Heart isolation followed previously established protocols [39]. The isolated hearts were promptly placed in ice-cold (4 ^∘^C) Krebs–Hensleit solution [40]. Cannulation and perfusion of the heart were performed as previously described [41]. A 30-min occlusion of the left anterior descending (LAD) coronary artery was conducted to induce regional ischemia. Preload was maintained at 6 mmHg, and perfusion pressure (PP) was consistently kept at 50 mmHg throughout the experimental procedures. PP was measured in a branch of the aortic cannula downstream of the flow probe using a Statham pressure transducer (P23 Db). Electronic control of constant PP was achieved using the perfusion assembly [“Module PPCM type 671 (Hugo Sachs Elektronik-Harvard Apparatus GmbH, Germany)”], allowing precise adjustment of PP between 5 and 150 mmHg, with an accuracy of ±1 mmHg.

The cationic G7 PAMAM dendrimer nanoparticles (nominally 8.1 nm in diameter, MW of 116,493, and bearing 512 surface amino groups) were synthesized by Dendritech (USA) and acquired from Sigma Chemical Company (St. Louis, MO, USA). Unless otherwise stated, all other reagents used in this study were obtained from Sigma Aldrich (St. Louis, MO, USA).

### Study protocol

**Figure 1.**
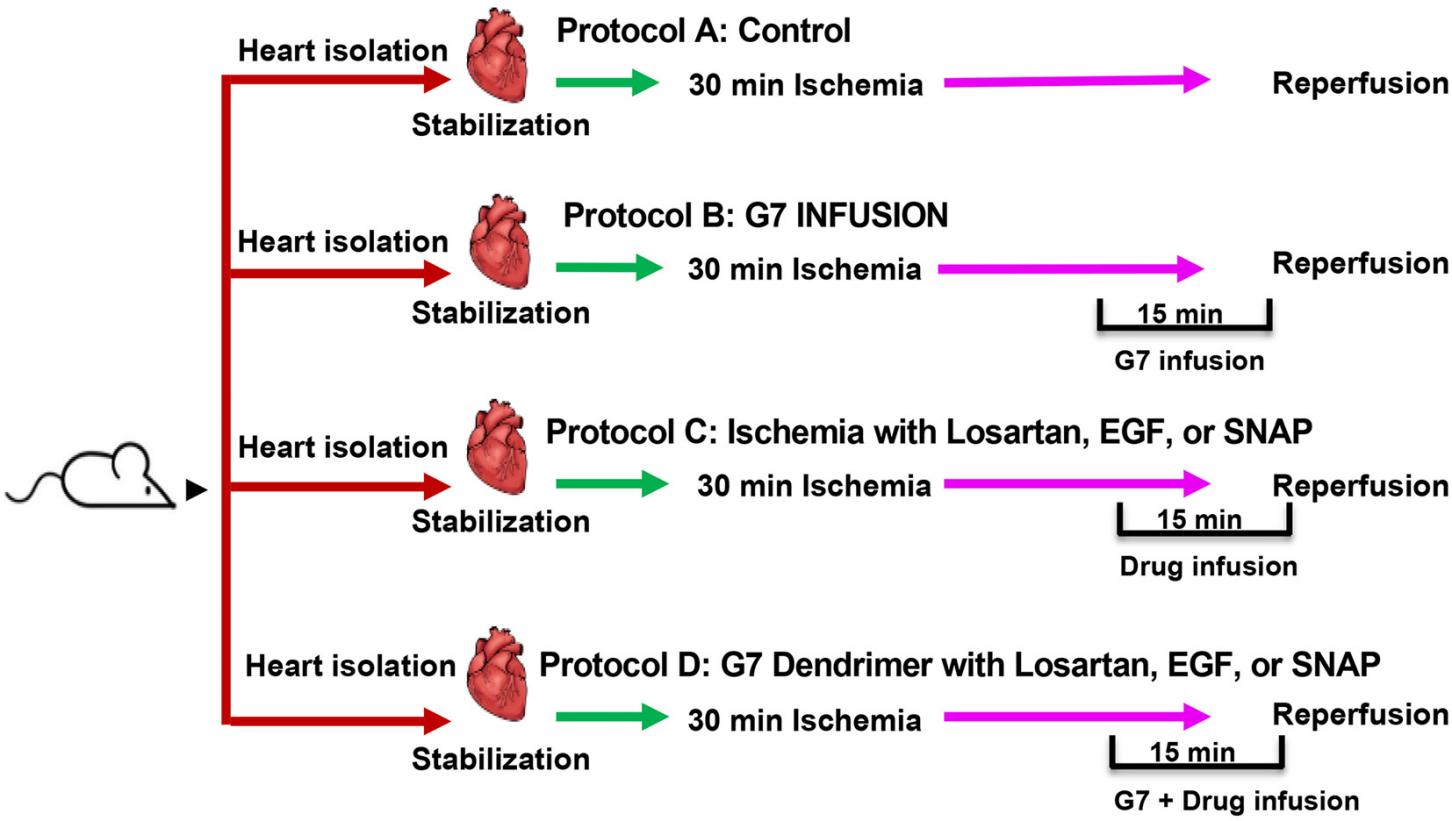
**Diagrammatic representation of the study groups and protocols used in the study.** Isolated rat hearts were divided into four main groups (*n* ═ 8), subjected to different experimental protocols labeled as protocols A, B, C, and D. Hearts underwent 30 minutes of ischemia followed by 30 min of reperfusion (I/R) (control) (Protocol A). I/R hearts were treated either with cationic G7 PAMAM dendrimer starting 5 min prior to and continuing for the first 10 min of reperfusion (Protocol B), or with Losartan, EGF, or SNAP commencing 5 min prior to and continuing for the first 10 minutes of reperfusion (Protocol C). Lastly, I/R hearts were treated with G7 PAMAM dendrimer in the presence of Losartan, EGF, or SNAP, starting 5 minutes prior to and continuing for the first 10 minutes of reperfusion (Protocol D). PAMAM: Polyamidoamine; EGF: Epidermal growth factor; SNAP: S-nitroso-N-acetylpenicillamine; I/R: Ischemia and reperfusion; G7: Seventh-generation cationic PAMAM dendrimer.

One set of hearts (*n* ═ 8 per group) underwent 30 min of regional ischemia followed by reperfusion, as previously described by Mohammad and Babiker [38]. Subsequently, all hearts were reperfused for an additional 30 min. Control hearts were subjected to I/R injury without any additional treatment. Another set of hearts subjected to ischemia received an infusion of 100-nM cationic G7 PAMAM dendrimer (refer to Figure 1). Alternatively, infusions of 1-µM Losartan, 10-nM EGF, or 1-µM SNAP, with or without 100-nM cationic G7 PAMAM dendrimer, were administered at the onset of reperfusion in the presence of ischemia. All treatments were initiated 5 min before reperfusion and continued for the initial 10 min of reperfusion (refer to Figure 1). Cardiovascular functions were determined as described previously [39, 42, 43]. Left ventricular (LV) contractility, hemodynamics, and coronary vascular dynamics were assessed throughout the experiment. LV dynamics were evaluated by measuring maximum developed pressure (DPmax), Left ventricular (LV) end-diastolic pressure (LVEDP), and LV contractility indices (+ dP/dt and −dP/dt). Coronary vascular dynamics were assessed by coronary vascular resistance (CVR) and coronary flow (CF). CF (mL/min) was monitored using an electromagnetic flow probe attached to the inflow of the aortic cannula, as previously described by Ismaeil et al. [44], and was digitally computed using software developed by Hugo-Sachs (Hugo-Sachs Elektronik, Germany). CF values were manually verified via the collection of coronary effluent over time. CVR and hemodynamic data were sampled every 10 s through an established data acquisition program (Hugo-Sachs’ Isoheart software V 1.524-S). Following the conclusion of each experiment, hearts were snap-frozen in liquid nitrogen and stored at −80 ^∘^C for further analysis.

### Measurements of infarct size and cardiac enzyme levels as indicators of cardiac injury

Infarct size was assessed using triphenyl tetrazolium chloride (TTC) staining, following established procedures [45]. Images were captured with a Nikon camera, and red and pale unstained areas on each slice were assessed using Leica ImageJ software (ImageJ, Wayne Rasband, and National Institute of Health, USA). The percentage of infarct area was determined relative to the total LV area. Cardiac injury was determined through the release of creatine kinase (CK) and lactate dehydrogenase (LDH) enzymes into the coronary effluent during reperfusion, as previously described [46].

### Data analysis

The acquired data were analyzed using two-way analysis of variance (ANOVA), followed by post hoc analysis with the least significant difference (LSD) method, utilizing SPSS software. Comparisons were made between group means and their respective controls. Results were expressed as mean ± standard error of the mean, with statistical significance considered at *P* < 0.05.

## Results

**Figure 2.**
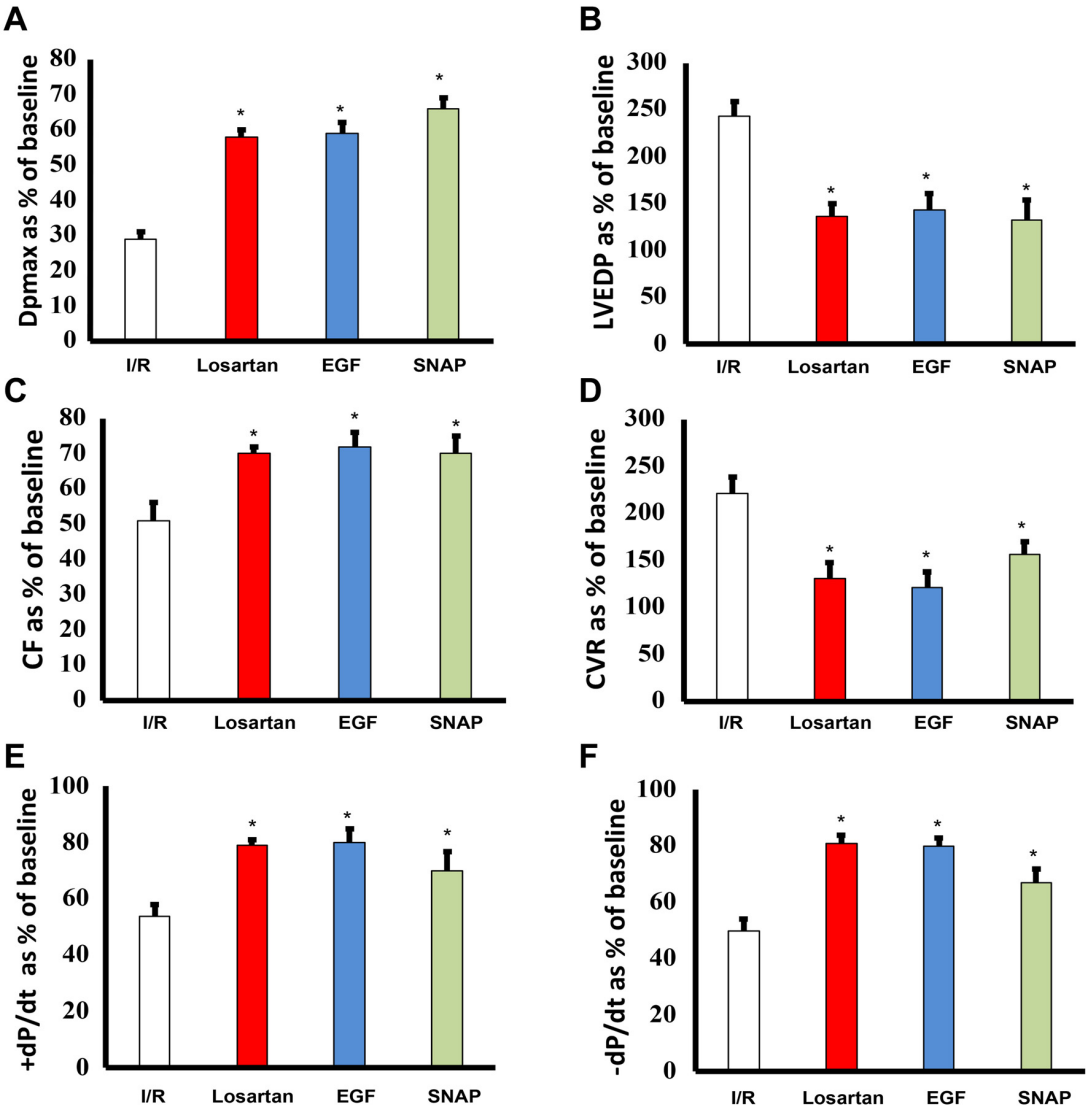
**I/R injury compromises recovery of cardiac function that is improved by treatment with Losartan, EGF or SNAP.** Post I/R recovery in the left ventricle function (DPmax (A) and LVEDP (B)), coronary vascular dynamics (CF (C) and CVR (D)) and contractility indices (+ dP/dt (E) and −dP/dt (F)) without and with treatment with Losartan, EGF, or SNAP is shown. N=8. Mean +/− SEM. Asterix indicates significant difference (*P* < 0.05) from I/R controls. LVEDP: Left ventricular (LV) end-diastolic pressure; SNAP: S-nitroso-N-acetylpenicillamine; EGF: Epidermal growth factor; CVR: Coronary vascular resistance; CF: Coronary flow; I/R: Ischemia and reperfusion.

In the animals used for this study, the mean body weight of the rats (295 ± 55 g) and heart size (1.45 ± 0.32 g) at sacrifice did not significantly differ among animal groups investigated. Regional cardiac ischemia for 30 min resulted in a significant (*P* < 0.05) deterioration in LV hemodynamics, contractility, and coronary vascular dynamics compared to baseline data (expressed as % of baseline; see Figure 2).

**Table 1:** Effects of I/R injury with or without treatments of Losartan, EGF, SNAP, and/or cationic G7 PAMAM dendrimer on cardiac enzyme levels

**Treatment**	**CK (IU/L)**	***P* value**	**LDH (IU/L)**	***P* value**
I/R	37.52±2.85	–	30.27±1.23	–
+ Losartan	30.13±2.42*	0.01	24.90±1.98*	0.01
+ EGF	29.53±1.72*	0.01	25.12±1.78*	0.01
+ SNAP	25.71±1.85*	0.001	22.21±1.73*	0.001
+ G7	48.61±2.54^*^	0.01	39.92±1.7^*^	0.01
+ G7 + Losartan	37.83±1.25**	0.02	29.87±2.16**	0.01
+ G7 + EGF	37.31±1.12**	0.01	29.17±1.28**	0.02
+ G7 + SNAP	35.51±1.27**	0.03	28.92±1.34**	0.01

Asterix (*) indicates values significantly different from I/R controls whereas double Asterix (**) indicates values significantly different from G7 treatment only in I/R injury (*P* < 0.05). LDH: Lactate dehydrogenase; CK: Creatinine kinase; PAMAM: Polyamidoamine; EGF: Epidermal growth factor; SNAP: S-nitroso-N-acetylpenicillamine; I/R: Ischemia and reperfusion; G7: Seventh-generation cationic PAMAM dendrimer.

To confirm their cardioprotective actions, we treated hearts subjected to I/R injury with Losartan, EGF, or SNAP (see Figure 1 for protocol). Infusion of any of these three agents 5 min before reperfusion, and continued for the first 10 min of reperfusion, significantly (*P* < 0.001) improved all measured cardiac function parameters (Figure 2) and reduced infarct size and cardiac enzyme levels for LDH and CK compared to I/R alone controls (Figure 4 and Table 1). For example, the LV function parameter DPmax improved approximately 2-fold for all treatments from around 30% for I/R alone to over 60% of baseline when treated with Losartan, EGF, or SNAP (Figure 2A). Similarly, recovery of the LV contractility indices (+ dP/dt and −dP/dt) upon treatment with the cardioprotective agents nearly doubled compared to I/R alone (Figure 2D and 2E). In contrast, LVEDP, which is markedly raised following I/R injury to around 250% of the baseline value, was reduced to half that value upon treatment with any one of the cardioprotective agents (Figure 2B). In terms of coronary vascular dynamics, treatment of hearts with either Losartan, EGF, or SNAP significantly increased CF by about 50% and reduced CVR to approximately half the values for I/R alone (Figure 2B and 2C), confirming the beneficial cardioprotective actions of all three agents in cardiac I/R injury.

**Figure 3.**
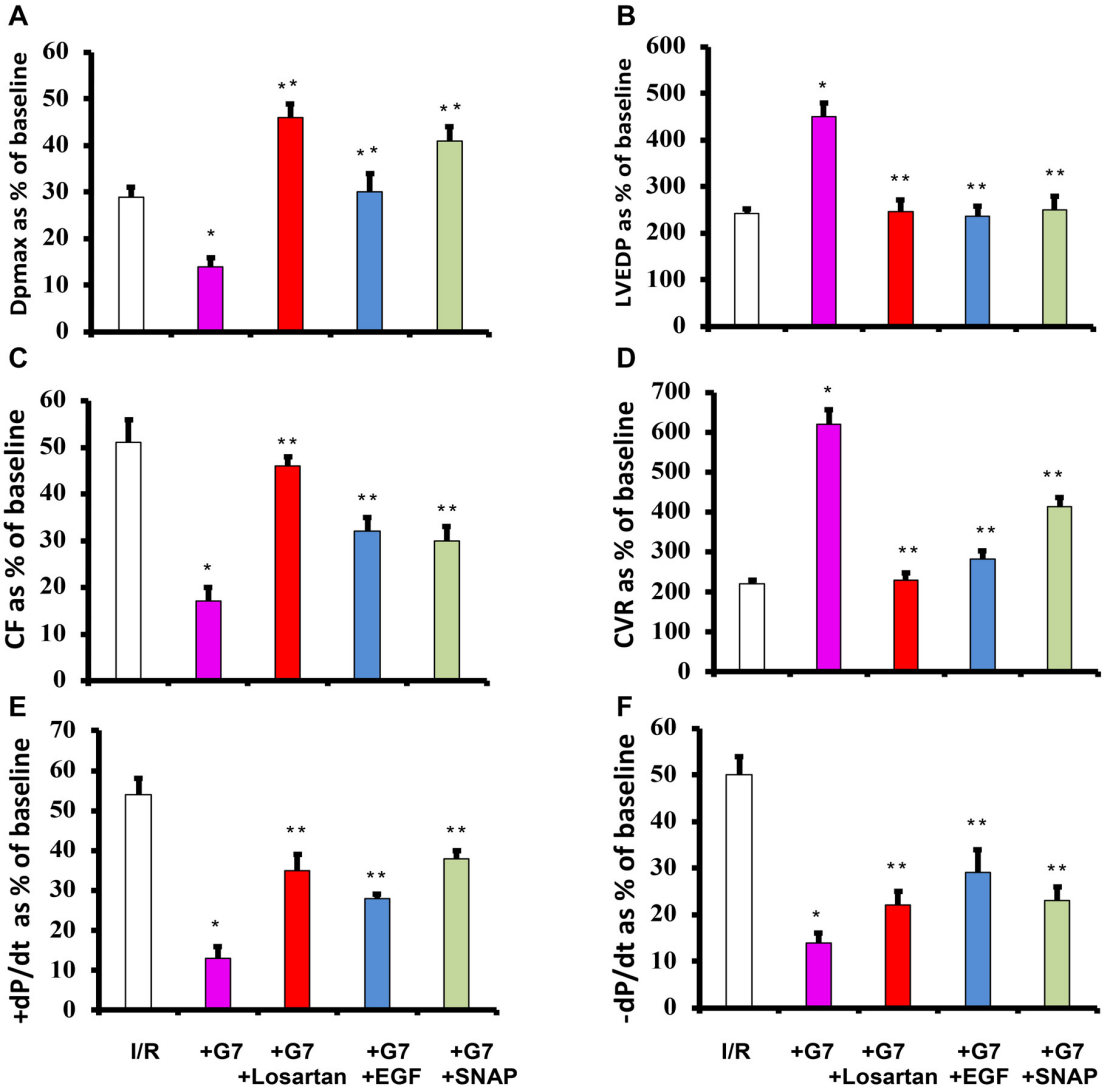
**Cationic G7 PAMAM dendrimer exacerbates cardiac function recovery from I/R injury that is rescued by co-administration of either Losartan, EGF or SNAP.** Post I/R recovery in the left ventricle function (DPmax (A) and LVEDP (B)), coronary vascular dynamics (CF (C) and CVR (D)) and contractility indices (+ dP/dt (E) and −dP/dt (F)) without and with treatment with G7 PAMAM dendrimer (G7) or G7 together with Losartan, EGF, or SNAP is shown. N=8. Mean +/− SEM. Asterix indicates significant difference (*p* < 0.05) from I/R controls. Asterix (*) indicates values significantly different from I/R controls whereas double Asterix (**) indicates values significantly different from G7 treatment only in I/R injury (*P* < 0.05). LVEDP: Left ventricular (LV) end-diastolic pressure; SNAP: S-nitroso-N-acetylpenicillamine; EGF: Epidermal growth factor; CVR: Coronary vascular resistance; CF: Coronary flow; PAMAM: Polyamidoamine; I/R: Ischemia and reperfusion; G7: Seventh-generation cationic PAMAM dendrimer.

**Figure 4.**
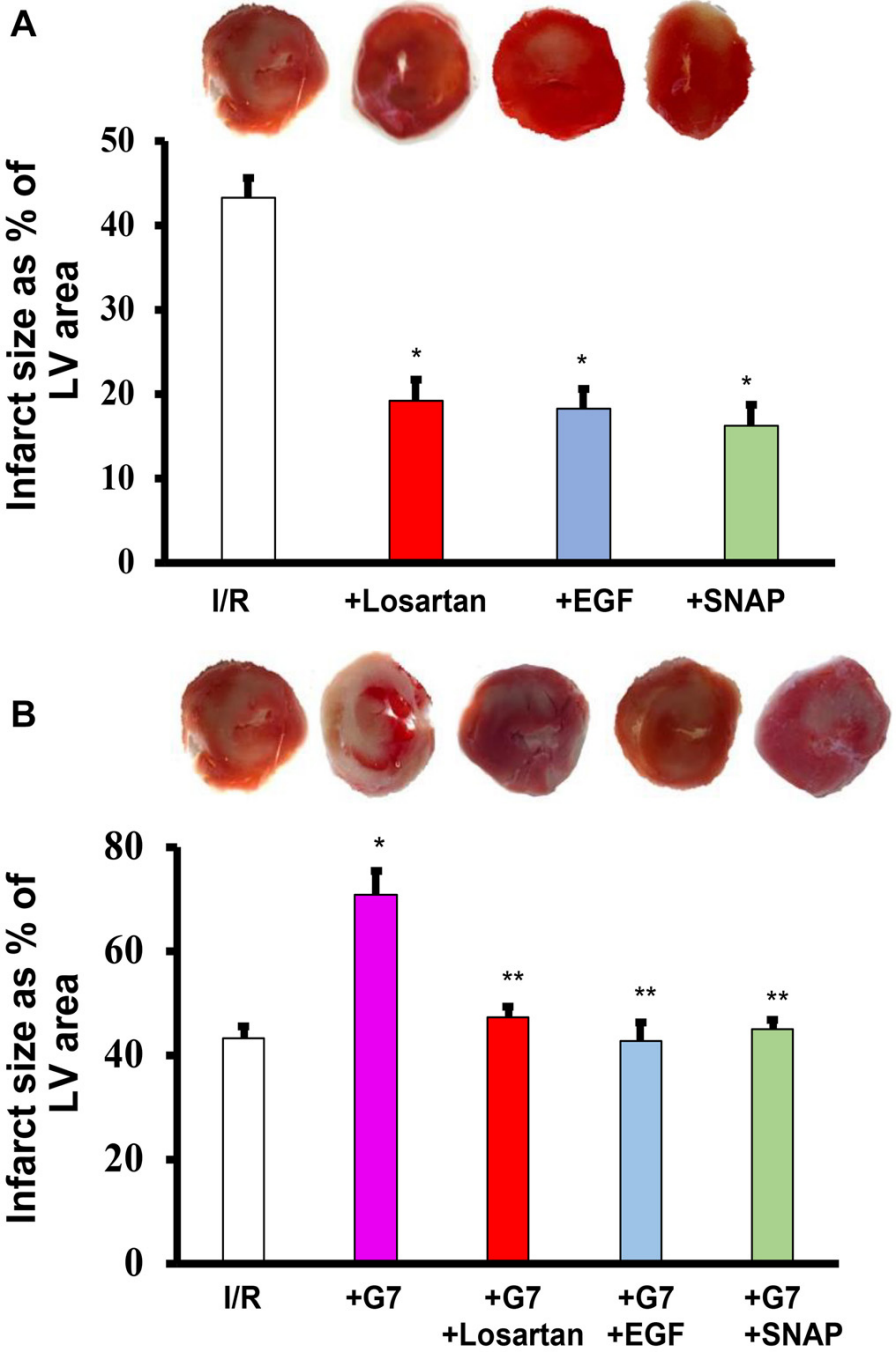
**Histological assessment of ischemic injury via determination of infarct size (expressed as infarcted area as a percentage of left ventricle area).** Panel A: Infarct size following I/R injury and upon treatment with Losartan, EGF, or SNAP 5min before and in first 10 min of reperfusion. Panel B: Infarct size following I/R injury, and I/R injury hearts treated with G7 PAMAM (+ G7) dendrimer and/or co-infused with Losartan, EGF or SNAP 5min before and in first 10 min of reperfusion. In both figure A and B, the top panel shows representative 2,3,5-triphenyl-2H-tetrazolium chloride-stained heart slices for each indicated experimental group. Asterix (*) indicates values significantly different from I/R controls whereas double Asterix (**) indicates values significantly different from G7 treatment only in I/R injury (*P* < 0.05). LVEDP: Left ventricular (LV) end-diastolic pressure; SNAP: S-nitroso-N-acetylpenicillamine; EGF: Epidermal growth factor; CVR: Coronary vascular resistance; CF: Coronary flow; PAMAM: Polyamidoamine; I/R: Ischemia and reperfusion; G7: Seventh-generation cationic PAMAM dendrimer.

Infusion of the G7 PAMAM dendrimer into isolated hearts significantly exacerbated LV hemodynamics, contractility, and coronary vascular dynamics parameters by 2- to 4-fold compared to hearts subjected to I/R alone (Figure 3). However, co-infusion of Losartan, EGF, or SNAP with G7 PAMAM dendrimer resulted in a significant improvement (*P* < 0.001) in DPmax, LVEDP, and LV contractility compared to G7 PAMAM dendrimer infusion alone (Figure 3). Similarly, coronary vascular dynamics, in terms of CF and CVR, which were markedly impaired by G7 PAMAM dendrimer infusion following I/R injury were significantly improved by the co-infusion of Losartan, EGF, or SNAP (*P* < 0.001) compared to G7 PAMAM treatment alone (Figure 3C and 3D). This recovery of cardiac function parameters was mirrored by data on infarct size and cardiac enzyme levels (Table 1). Indeed, adjunct treatment with Losartan, EGF, or SNAP significantly (*P* < 0.01) mitigated the G7 PAMAM dendrimer-induced increase in infarct size and cardiac CK and LDH enzyme levels (Figure 4 and Table 1).

## Discussion

The structurally defined and highly versatile, polyvalent PAMAM series of dendrimer nanoparticles are increasingly being considered for drug delivery and other potential clinical applications in nanomedicine [1, 2]. However, their full safety and toxicological profile, especially in target organs like the mammalian heart, are not fully understood. We previously showed that *ex vivo* and *in vivo* administration of cationic PAMAM dendrimers can compromise cardiac function recovery in mammalian hearts following I/R injury [16, 17]. The extent of PAMAM dendrimer-induced cardiac dysfunction depends on the physicochemical properties of the nanoparticles, such as their molecular size, weight (generation), and surface-group chemistry [16]. Generally, higher generation/molecular weight PAMAMs (e.g., G6 and G7) with cationic surface chemistry (amino groups) exhibit the greatest cardiac toxicity [16]. However, since higher generation PAMAMs also enhance cellular uptake and delivery properties for drugs, such as nucleic acid-based gene silencing and gene-editing therapeutics [1, 2], strategies to overcome their adverse cardiac effects will likely be required for clinical use. Although several strategies for decreasing the general cellular toxicity of PAMAMs are possible, such as PEGylation or reducing surface cationic charge density (see [2] for a recent review), the direct effects of such modifications on heart function are not known.

As an alternative approach, we previously reported that adjunct use of the cardioprotective agent ang-(1–7), a heptapeptide member of the RAS, could mitigate the cardiotoxicity of a cationic G7 PAMAM dendrimer [16]. In this study, we extended these findings to investigate whether other known cardioprotective agents could rescue the adverse cardiac effects of a cationic G7 PAMAM dendrimer in the mammalian heart. The major findings of this study are that adjunct administration of any of three different cardioprotective agents—Losartan, EGF, or SNAP—can significantly rescue the adverse cardiac effects of a cationic G7 PAMAM dendrimer in the isolated mammalian heart following I/R injury. Thus, the adjunct use of such general cardioprotective agents might be a useful strategy for mitigating the cardiotoxicity of PAMAM dendrimers and, potentially, other drug delivery systems *in vivo.*

Cationic PAMAM dendrimers are known to biodistribute to the heart (along with other organs of the reticuloendothelial system) following systemic administration [15, 47] and have been shown to preferentially accumulate within ischemic regions of the myocardium [15]. This suggests that passive targeting of ischemic myocardial tissue is possible with PAMAM delivery systems but also indicates that the ischemic heart may be more vulnerable to adverse effects from charged PAMAM dendrimers compared to healthy heart tissue. Although the precise mechanisms by which cationic PAMAM dendrimers compromise cardiac function are not fully understood, we have previously proposed that they may interfere with key recovery or salvage pathways activated in the heart during I/R injury and/or physically occlude coronary vasculature due to their nanoparticulate nature, reducing CF and increasing CVR [16, 17]. While both mechanisms are likely involved, given that PAMAMs exhibit vasculoprotective effects and prevent vascular dysfunction in an animal model of diabetes [8], it is plausible that they primarily attenuate one or more cardiac survival signaling cascades that aid recovery from I/R injury.

We have previously identified that activation of EGFR signaling via the PI3K/AKT pathway is critical for cardiac function recovery in I/R injury, representing a key salvage pathway in the ischemic heart [22]. Additionally, we have shown that naked cationic PAMAM dendrimers are effective inhibitors of EGFR signaling cascades [5, 6, 8]. Thus, it is tempting to speculate that cationic G7 PAMAM dendrimer-induced impairment in cardiac recovery following I/R injury occurs via the blockade of the key survival pathway mediated by EGFR/P13K/AKT signaling. This is consistent with our findings in this study, where cardioprotective EGF, a ligand for EGFR known to activate the EGFR/P13K/AKT salvage pathway in ischemic hearts [22], mostly abrogated the adverse cardiac effects of the G7 PAMAM dendrimer. Indeed, we have previously shown that cardiac EGFR signaling is attenuated following ischemia [22], leading to impaired recovery from I/R injury, as seen in this study (Figures 2 and 3). The fact that G7 dendrimer treatment further exacerbated cardiac recovery in all measured cardiac function parameters (Figure 3) is consistent with further inhibition of EGFR signaling by G7 PAMAM dendrimer and subsequent attenuation of the EGFR-mediated salvage pathway necessary for cardiac function recovery. Rescue of ischemia and/or G7 dendrimer-mediated attenuation of cardiac EGFR signaling by exogenous EGF administration would explain the improved recovery from I/R injury (Figures 2 and 3), though further study is needed. Additionally, EGF/EGFR signaling can impact cardiovascular function in various other ways, including through modulation of ROS formation, NO production, and regulation of myocardial apoptosis (for a recent review, see [25]). This may also explain the reduction in infarct size and cardiac enzyme levels observed in this study upon EGF infusion following ischemic injury (Table 1 and Figure 4). Thus, adjunct use of EGF appears to be a promising strategy for mitigating dendrimer-induced cardiotoxicity. This could be achieved through co-administration of EGF or by conjugating EGF to PAMAM dendrimers, as has been reported for targeting cancer cell surfaces [49, 50].

**Figure 5.**
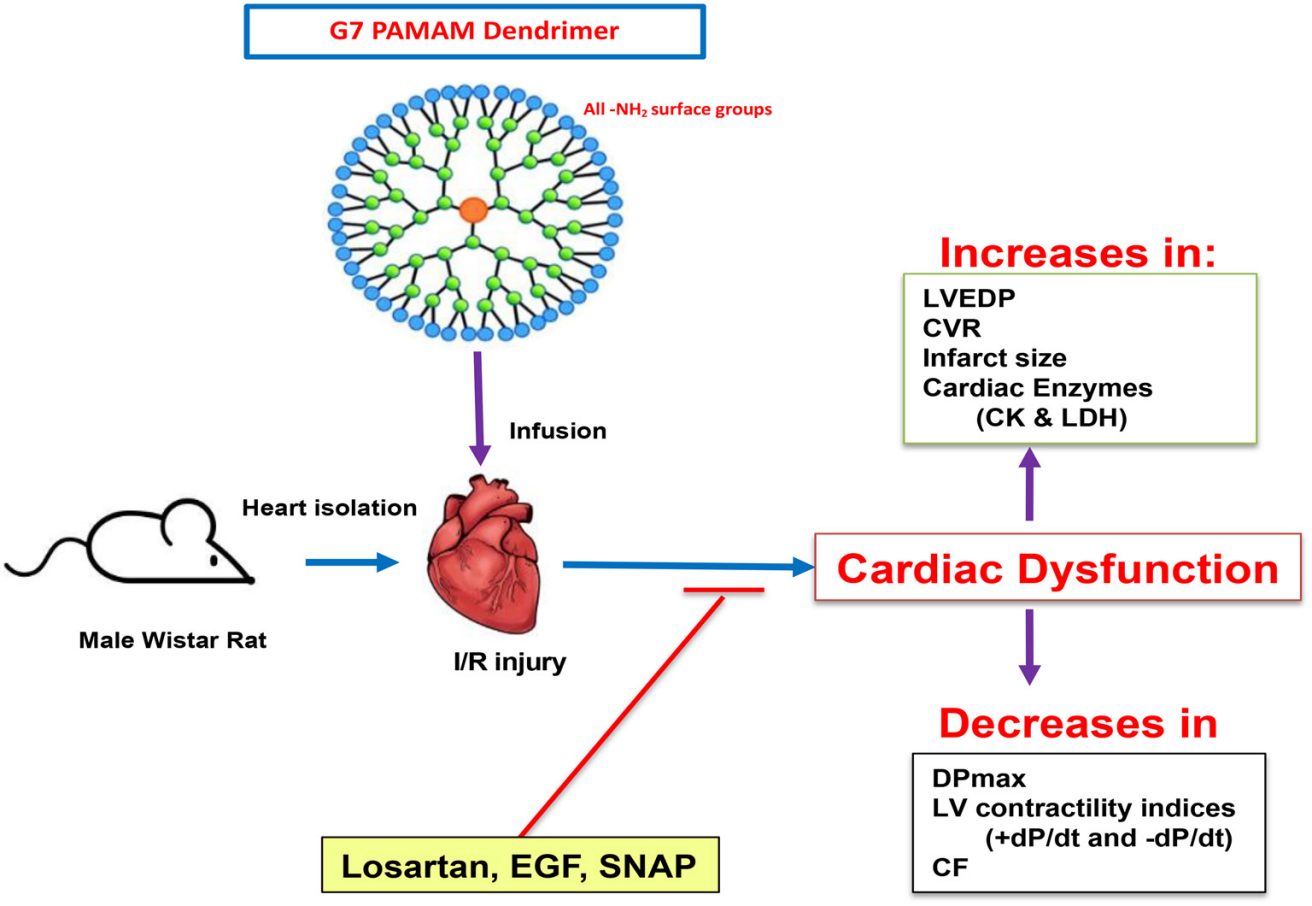
**Cardiac effects of cationic G7 PAMAM dendrimer and the ability of cardioprotective agents to abrogate their cardiotoxicity.** Cardiac administration of G7 PAMAM dendrimer bearing all amino (-NH2) surface groups impaired post-I/R recovery of hearts by increasing LVEDP, CVR, cardiac enzyme levels (LDH, CK), infarct size and decreasing DPmax, LV contractility indices (+ dP/dt and −dP/dt) and CF, all indicative of cardiac dysfunction and myocardial injury. These adverse cardiac effects of G7 PAMAM dendrimer were markedly attenuated by adjunct administration of the cardioprotective agents, Losartan, EGF or SNAP. Thus, adjunct use of these cardioprotective agents may represent a viable pharmacological strategy to mitigate or abrogate the cardiotoxicity of PAMAM dendrimers as well as potentially other polymeric nanoparticulate drug delivery systems. LVEDP: Left ventricular (LV) end-diastolic pressure; SNAP: S-nitroso-N-acetylpenicillamine; EGF: Epidermal growth factor; CVR: Coronary vascular resistance; CF: Coronary flow; CK: Creatine kinase; LDH: Lactate dehydrogenase; LV: Left ventricular; PAMAM: Polyamidoamine; G7: Seventh-generation cationic PAMAM dendrimer.

Losartan is a well-established cardiovascular drug with an acceptable safety profile that has been on the market since 1995 and is clinically approved for the treatment of cardiovascular disorders, including hypertension, diabetic nephropathy, and stroke [51]. Consistent with the findings of our present study (see Figure 2), it has previously been shown by us and others that Losartan is also cardioprotective in I/R injury in most studies reported in the literature, though not all [20–24, 40, 52, 53]. Although its precise effects in I/R injury require further clarification, in the present study, Losartan infusion markedly improved recovery from I/R injury across all cardiac parameters studied (namely DPmax, LVEDP, CF, CVR, and the contractility indices +dP/dt and −dP/dt), implying it has broad cardioprotective properties that impact both cardiac contractility and coronary hemodynamics. As a blocker of AT1R (termed ARB), Losartan opposes the detrimental effects of Angiotensin II (Ang II) on the heart, which classically lead to increased formation of ROS, vasoconstriction, and apoptosis, among others (see [19, 25, 52]). The fact that we also observed a reduction in infarct size and cardiac enzymes with Losartan treatment (Figure 4 and Table 1) might be explained by its ability to reduce Ang II-induced myocardial apoptosis. Some of the beneficial effects of Losartan might also be mediated in part by increasing endogenous Ang-(1–7). It has been shown that ACE inhibitors and ARBs increase Ang-(1–7) formation in the heart, and Ang-(1–7) receptor antagonists attenuate their cardiovascular benefits [54–56]. Ang-(1–7) is a coronary vasodilator and has anti-inflammatory, antioxidant, and antithrombotic effects [54, 57–59]. Ang-(1–7) has been shown to decrease I/R-induced calcium overload and ROS production, leading to cardioprotective effects [60]. Interestingly, Ang-(1–7) potentiates bradykinin-induced vasodilation of porcine coronary arteries by acting as an ACE inhibitor [61]. Chang et al. showed that Ang-(1–7) protects cardiomyocytes from long-term hypoxia-stimulated apoptosis. Indeed, Ang-(1–7) can mitigate G7 PAMAM-mediated cardiac dysfunction in I/R injury [16]. Notably, Losartan also ameliorated lung injury caused by a PAMAM dendrimer [62]. Despite its already significant cardioprotective effects, Losartan’s benefits can be significantly enhanced by co-administration of EGF [22]. This is thought to be because EGF can rescue the partial Losartan-induced reduction in otherwise beneficial cardiac EGFR signaling by blocking Ang II-mediated transactivation of EGFR [22]. Though not attempted in the current study, a combination of Losartan and EGF (or other cardioprotective agents) might afford greater protection against G7 PAMAM-induced cardiotoxicity, though this requires further research and consideration of any potential drug-drug interactions.

Losartan and EGF are both known to increase NO production, most likely through increased eNOS activity [22, 63, 64], which is an important regulator of microvascular flow and exhibits known cardioprotective actions in the heart following I/R injury [29, 30]. Thus, the mechanisms by which Losartan and EGF exert their cardioprotective effects might converge at the level of NO. Indeed, this notion is supported by our data, which show a similar magnitude of cardiac recovery in the different cardiac contractility and hemodynamic parameters assessed for each of these two agents and the NO donor, SNAP (see Figures 2 and 3). Although NO is a potent vasodilator with the potential to attenuate cardiac I/R injury, it can also mediate tissue injury, but this appears to be concentration dependent [37]. By using a previously defined 1 µM dose of SNAP, which is known to be cardioprotective and has been shown to reduce infarct size in rodent hearts subjected to ischemia (37, 38), we found that infusion of this NO donor improved all cardiac parameters studied and reduced infarct size and cardiac enzyme levels due to I/R (Figures 2 and 4; Table 1), as well as the further exacerbation of I/R injury with G7 PAMAM dendrimer (Figures 3 and 4; Table 1). These data confirm that, in our hands, the administration of exogenous NO at the right dose can protect heart function and reduce cardiac injury and myocardial infarction. Furthermore, these results suggest that increasing NO levels with SNAP might be a useful strategy to reduce G7-mediated cardiotoxicity. Indeed, conjugates of a G4 PAMAM and SNAP have been reported [65, 66], with one study showing that using a glutathione-initiated release of NO from these dendrimers could reduce I/R injury [65], which supports our report of using free (unconjugated) SNAP as a strategy to rescue PAMAM-mediated toxicity. Additionally, the potential co-administration of other cardioprotective drugs, either as free drugs or associated with PAMAM delivery systems (e.g., either entrapped within or covalently conjugated to the surface of nanoparticles), might provide similar cardioprotection to that observed in this study with free Losartan, EGF, and SNAP. Indeed, a PAMAM dendrimer conjugated to an agonist of the A3 adenosine receptor, which activates key recovery pathways in hearts, improved cardiac function recovery and reduced infarct size following I/R injury [67, 68]. Thus, by using appropriate dendrimer-drug combinations or through simple adjunct administration of “free” drugs known to exert cardioprotective actions, such as Losartan, EGF, or SNAP, as reported here (see Figure 5 for a summary), or even Ang-(1–7) shown by us previously [16], the cardiotoxicity of PAMAM dendrimers might be abrogated. Indeed, such cardiotoxicity-rescuing strategies might be necessary to improve the safety profile of PAMAM dendrimers for potential clinical use. As to whether this strategy will be effective in mitigating the cardiotoxicity of other dendrimer drug delivery systems, such as polypropylyimine (PPI) and polyethyleneimine (PEI), is not yet known. Studies investigating the direct cardiotoxicity of these dendrimer-based delivery systems are sparse. However, we have previously shown that PPI dendrimers elicit multiple gene expression changes in cells [69] and are known to exhibit neurotoxicity [70]—changes that could potentially also modulate cardiac function. A common feature of the aforementioned dendrimers is their polycationic nature, which is likely the cause of cellular toxicity *in vitro* and *in vivo* [2]. Although not studied, it is quite likely that PAMAM nanoparticles and related polycationic delivery systems will also exhibit toxicity via similar cellular mechanisms in the heart [2, 16, 17]. If confirmed, the use of diverse cardioprotective agents, such as clinically acceptable ARBs, NO donors, and growth factors, as reported here for PAMAMs, might also be effective in mitigating the cardiotoxicity of these related delivery systems.

## Conclusion

PAMAM-induced cardiac toxicity could be significantly attenuated by the adjunct administration of well-established cardioprotective drugs such as Losartan, EGF, or SNAP (see Figure 5). Thus, the co-administration of these pharmacological agents might represent a novel strategy to rescue or prevent the cardiotoxicity elicited by PAMAM dendrimers and potentially other nanoparticle-based drug delivery systems.

## Data Availability

The data presented in this study are available on request from the corresponding authors.
